# Effect of Low Nesquehonite Addition on the Hydration Product and Pore Structure of Reactive Magnesia Paste

**DOI:** 10.3390/ma16062445

**Published:** 2023-03-18

**Authors:** Run Shi, Yuehan Hao, Deping Chen, Wenxin Liu

**Affiliations:** Beijing Key Laboratory of Urban Underground Space Engineering, School of Civil and Resource Engineering, University of Science and Technology Beijing, Beijing 100083, China

**Keywords:** nesquehonite, reactive magnesia, Mg(OH)_2_, magnesium carbonate hydroxide, pore structure, nanopore

## Abstract

Reactive magnesia cement is considered an eco-efficient binder due to its low synthesis temperature and CO_2_ absorption properties. However, the hydration of pure MgO–H_2_O mixtures cannot produce strong Mg(OH)_2_ pastes. In this study, nesquehonite (Nes, MgCO_3_·3H_2_O) was added to the MgO–H_2_O system to improve its strength properties, and its hydration products and pore structure were analyzed. The experimental results showed that the hydration product changed from small plate-like Mg(OH)_2_ crystals to interlaced sheet-like crystals after the addition of a small amount of Nes. The porosity increased from 36.3% to 64.6%, and the total pore surface area increased from 4.6 to 118.5 m^2^/g. At the same time, most of the pores decreased in size from the micron scale to the nanometer scale, which indicated that Nes had a positive effect on improving the pore structure and enhancing the compressive strength. Combined with an X-ray diffractometer (XRD), a Fourier transform infrared spectrometer (FTIR), and a simultaneous thermal analyzer (TG/DSC), the hydration product of the sample after Nes addition could be described as xMgCO_3_·Mg(OH)_2_·yH_2_O. When Nes was added at 7.87 and 14.35 wt%, the x-values in the chemical formula of the hydration products were 0.025 and 0.048, respectively. These small x-values resulted in lattice and property parameters of the hydration products that were similar to those of Mg(OH)_2_.

## 1. Introduction

The cement industry is a serious source of fuel consumption and greenhouse gas (GHG) emissions; it is responsible for 5% of GHG emissions and is among the top industrial sources of carbon dioxide (CO_2_) emissions [1]. Various efforts have been made worldwide to reduce the cement industry’s large CO_2_ emissions, with the goal of improving the overall cost-effectiveness of energy use [2,3,4]. Compared with Portland cement clinker, reactive magnesia cement (RMC) is considered an eco-efficient binder due to its low synthesis temperature and CO_2_ absorption [5,6,7,8,9]. RMC is an emerging material whose ability to mineralize CO_2_ makes it useful for reducing the life-cycle global warming intensity (GWI) of concrete. Reactive MgO can be obtained through the calcination of MgCO_3_ and precipitation of Mg(OH)_2_ from seawater or brine [6,10,11]. Depending on the calcination temperature, four different grades of MgO can be produced: lightly fired (700–1000 °C), hard-fired (1000–1500 °C), dead-fired (1500–2000 °C), and molten (>2800 °C) [12,13,14]. Due to the loss of CO_2_ gas or water during calcination, lightly fired MgO has a porous structure and large surface area, making it highly reactive compared with other forms of MgO [15,16,17]. The MgO hydration product Mg(OH)_2_ has a very high heat absorption capacity and releases non-toxic water vapor during the dehydration process. The dehydrated product is a refractory material with a melting temperature of about 2800 °C [18,19], and Mg(OH)_2_-based materials offer great potential for fire protection applications in underground structures [20,21].

However, the hydration of a pure MgO–H_2_O mixture will not lead to a strong Mg(OH)_2_ paste. Studies have shown that the hydration of MgO can be significantly improved under additive and CO_2_ curing conditions [22,23,24,25,26,27]. The main products of MgO hydration consist of dypingite (Dyp, Mg_5_(CO_3_)_4_(OH)_2_·5H_2_O), nesquehonite (Nes, MgCO_3_·3H_2_O), and Mg(OH)_2_. The use of hydrated magnesium carbonate (HMC) additives, such as hydromagnesite [Hmgs, Mg_5_(CO_3_)_4_(OH)_2_·4H_2_O] [24,28], Nes [25], and Dyp [16], allows for the production of high-strength pastes without CO_2_ curing conditions. Kuenzel et al. [28] found that the compressive strength of MgO–H_2_O pastes with Hmgs additives at MgO: Hmgs mass ratios of 9:1, 8:2, and 7:3, and a water-to-binder ratio of 0.62 all reached values above 23 MPa after 28 days of curing in deionized water. The hydration product was mainly Mg(OH)_2_ with low crystallinity. HMCs can promote the hydration of MgO and improve the strength of the pastes. However, the composition of the hydration products and the pore structure characteristics of these pastes have not been well studied, and only the strength data have been reported for Nes additives [25]. In addition, as a CO_2_ sequestration product, Nes can be prepared from magnesium-oxide-rich minerals and rocks, mining and metallurgical solid wastes, and seawater at room temperature and atmospheric pressure [29,30,31,32].

In this study, the hydration mechanism, hydration product characteristics, and pore structure characteristics during the hardening and curing process were investigated using a field emission scanning electron microscope (FESEM), mercury intrusion porosimetry (MIP), an X-ray diffractometer (XRD), a simultaneous thermal analyzer (TG-DSC), and Fourier transform infrared spectroscopy (FTIR) analysis using a reactive MgO hybrid cementitious material with added Nes as the object of study. This provided an important basis for an in-depth study of the hydration mechanism of MgO in the presence of HMCs.

## 2. Materials and Methods

### 2.1. Materials

The reactive MgO used in this study was obtained from Shanghai Macklin Biochemical Co., Ltd., Shanghai, China, and consisted of an AR-grade chemical reagent of lightly burned MgO with particle diameter distributions of d90, d50, and d10, which were measured as 7.4, 3.3, and 1.3 μm, respectively.

**Figure 1:**
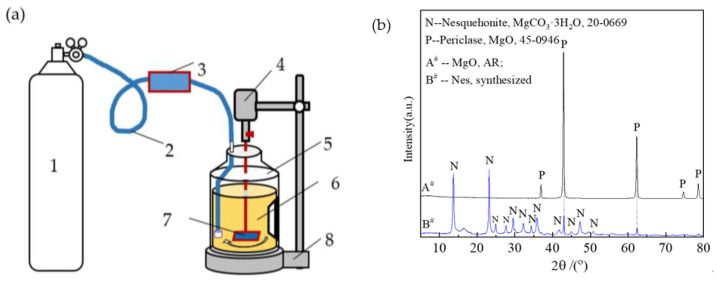
Diagram of Nes preparation apparatus: (**a**) schematic (1—CO_2_ gas cylinder; 2—hose; 3—CO_2_ flow controller; 4—mixing motor; 5—slurry drum; 6—slurry; 7—mixing blades; 8—mixer stand); (**b**) the diffraction pattern of MgO and Nes.

Nesquehonite (Nes) was synthesized using the carbonation of a reactive MgO suspension at room temperature. The experimental setup is shown in Figure 1a. First, 200 g of MgO was dispersed in 10 kg of deionized water to form a suspension. The flow rate of the CO_2_ gas was set to 0.8 L/min, the suspension was stirred at 500 rpm with a mixer until the pH value decreased to 7.8, and then it was allowed to stand for 15 min. After the reaction, the slurry was filtered, and the solid was placed in a blower drying oven and dried at 60 °C for 2 days. Figure 1b is the diffraction pattern of MgO and synthetic Nes. According to XRD and thermal gravimetric analyses, the dry solid contained 81.25% Nes by mass and 18.75% MgO residue by mass. The dry solid was pulverized into a powder, which particle diameter distributions of d90, d50, and d10, which were measured as 59.4, 11.5, and 2.9 μm, respectively.

The deionized water used was purchased from Shanghai Aladdin Biochemical Technology Co., Ltd., Shanghai, China.

### 2.2. Paste Preparation and Curing

**Figure 2:**
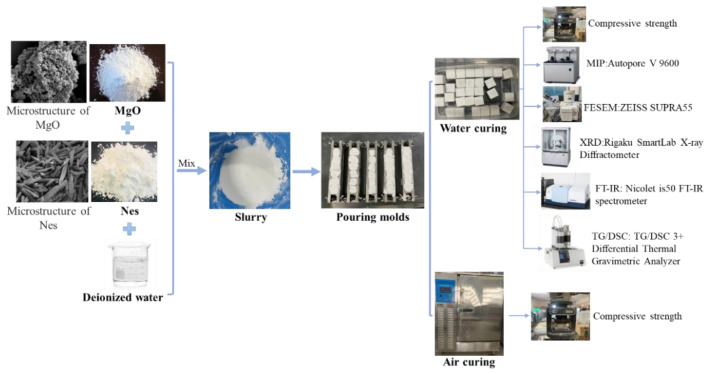
Experimental flow (weighing raw materials, mixing and casting of the slurry, and curing and testing of the samples).

The studied reactive MgO and synthesized Nes powder were mixed at MgO:Nes molar ratios of 1:0, 64:1, 32:1, and 16:1, and the actual mass fractions of the Nes additive in the MgO–Nes mixtures were 0%, 4.14%, 7.87%, and 14.35%, respectively (Table 1). Deionized water was added to form pastes without any other chemical agents, and the water-to-solid ratio used in all samples was 1.1, which was the minimum water content required to form a homogenous paste in all cases. The fresh pastes were cast into 20 mm × 20 mm × 20 mm cubic molds, vibrated for 5 min to remove air bubbles, and then covered with a plastic film to retain moisture and isolate from atmospheric CO_2_. The samples were then hydrated in a cement-curing chamber at 20 ± 1 °C for 24 h. The film was then removed, and the samples were immersed in deionized water at 20 ± 1 °C for 3, 7, and 28 days (referred to as “water curing”). For comparison, some samples were maintained at 20 ± 1 °C and relative humidity above 90% until the corresponding age (denoted as “air curing”). The flow of the experiments is shown in Figure 2.

### 2.3. Testing Methods

#### 2.3.1. Compressive Strength Test

The cured pastes were removed at a certain period from the water bath and chamber and tested in uniaxial compression at a loading rate of 50 N/s to obtain compressive strength.

#### 2.3.2. Pore Size Distribution and Total Pore Volume Test

Sample pieces 5–10 mm in size were submitted to a pore structure test by mercury intrusion porosimetry (MIP) using an Autopore V 9600 porosimeter (Micromeritics Instruments Co., Norcross, GA, USA). The samples were dried at 60 °C for 2 days before testing.

#### 2.3.3. Morphology of the Hydration Product and Pore Structure Observations

Field emission scanning electron microscope (FESEM) observations were conducted for the characterization of the cured pastes using a Zeiss Supra55 scanning electronic microscope (Carl Zeiss Microscopy LLC, White Plains, NY, USA). A thin gold layer was deposited on the samples to create a conductive surface for analysis.

#### 2.3.4. Hydration Product Analyses

The dried samples were crushed and manually ground with an alumina mortar and pestle. Their phase compositions were examined by XRD using a SmartLab diffractometer (Rigaku Co., Tokyo, Japan), with Cu Kα radiation in the range of 5–80° with a step size of 0.02°. Additional phases in the reaction products were detected by Fourier transform infrared spectroscopy (FT-IR) with a Nicolet is50 FT-IR spectrometer in the spectral range of 400–4000 cm^−1^.

#### 2.3.5. Weight Loss and Heat Absorption Capacity Test

The powder samples were submitted for thermal analysis in a TG/DSC 3+ thermogravimetry analyzer (Mettler-Toledo Inc., Columbus, OH, USA), which allowed for simultaneous TG and DSC analysis. Sample powders of approximately 33 mg were heated from room temperature to 1000 °C at a heating rate of 10 °C/min in N_2_. The weight loss and heat absorption of dehydration were calculated from the TG/DSC data.

## 3. Results

### 3.1. Compressive Strength

**Figure 3:**
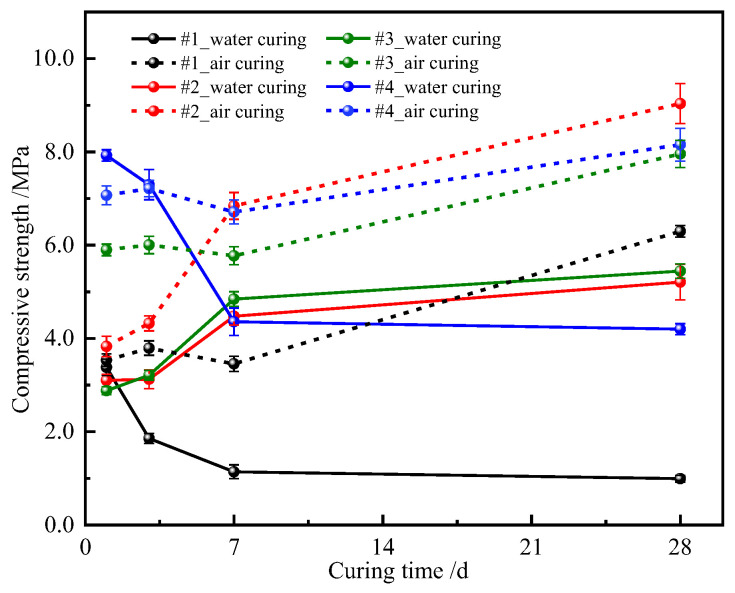
Compressive strength vs. curing time of the hydrated samples.

Figure 3 shows the variations in compressive strength of the hydrated samples with Nes ratio, curing conditions, and time. When the curing condition was water curing, for sample #1 with pure MgO, the compressive strength decreased with increased curing age and fell below 1 MPa, and then leveled off when the curing age was greater than 7 days. The compressive strength values of samples #2 and #3 increased with increased curing age and stabilized after 7 days. The compressive strength trend of sample #4 was similar to that of #1, but the overall strength was higher than that of sample #1, and the compressive strength remained between 3.7 and 7.9 MPa. Unlike water curing, the development of compressive strength of the air-cured samples showed an increasing trend, and the strength of samples added with Nes was higher than that of #1 samples with pure MgO. Among them, sample #2 reached a maximum strength of 9.0 MPa after 28 days of air curing. Therefore, we found that the introduction of Nes into the MgO-H_2_O system, whether cured in air or in water, tended to increase the compressive strength of the MgO paste for a given addition amount.

### 3.2. Pore Structure Characteristics

**Figure 4:**
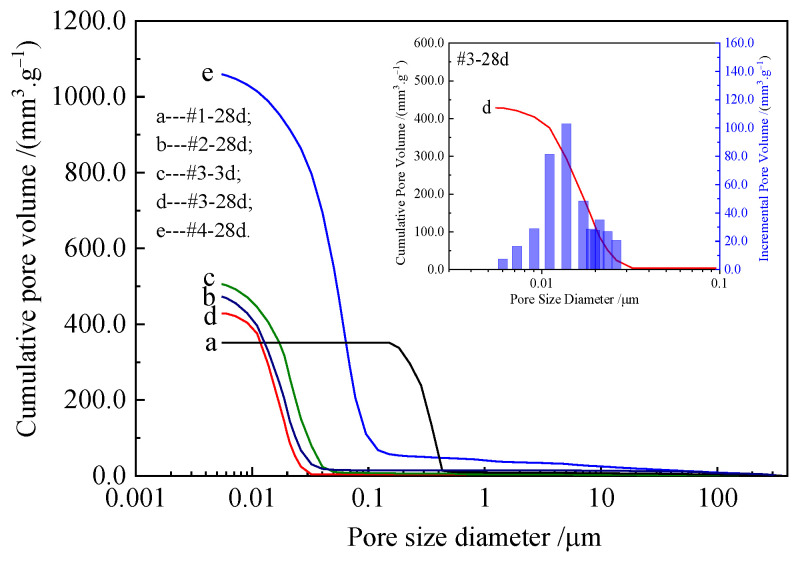
Cumulative pore size distribution curve of the hardened pastes. (**a**) #1-28d, (**b**) #2-28d, (**c**) #3-3d, (**d**) #3-28d, and (**e**) #4-28d.

The cumulative pore volume distribution curves of the water-cured samples are shown in Figure 4. For the 28-day-aged sample (#1-28d) consisting of pure MgO, the total pore volume was 351.3 mm^3^/g, of which 97.11% of the pore diameters were distributed between 150 and 500 nm. Meanwhile, without pores smaller than 150 nm, the pore volume of the pores above 500 nm accounted for only 2.89% of the total volume (Figure 4a), with an average pore diameter of 303.5 nm and total pore area of 4.6 m^2^/g. Compared with the pure MgO samples, the samples with less Nes additive had a larger total pore volume and finer pore diameters, mostly consisting of nanopores, and the total pore area increased. At the same curing time of 28 days, the curves of samples #2 and #3 were similar (Figure 4b,d), and the total pore volume, total pore area, and average pore diameter were similar. Among them, 97.30% of the pore diameters of sample #3 were below 30 nm, with an average pore size of 15.2 nm and a total pore area of 112.7 m^2^/g. Meanwhile, sample #4, with the 14.35% Nes addition (Figure 4e), had a much higher cumulative pore volume of 1059.6 mm^3^/g. The average pore diameter was 35.8 nm. As the amount of Nes added increased, the dry bulk density decreased.

The pore structure parameters of the samples are listed in Table 2. Table 2 shows that the dry bulk density of the samples of the same age decreased with an increase in the Nes addition and that the porosity increased. The bulk density and porosity of the pure MgO (#1-28d) samples were 1032 kg/m^3^ and 36.25%, respectively, and those of the #4-28d samples with 14.35% addition of Nes changed significantly to 609 kg/m^3^ and 64.55%, respectively. We observed that the addition of a small amount of Nes decreased the pore diameter and increased the total pore area of the samples. It should be noted that the same changes were found for sample #3 with a shorter 3-day curing time.

### 3.3. Morphology of the Hydration Products and Pores

**Figure 5:**
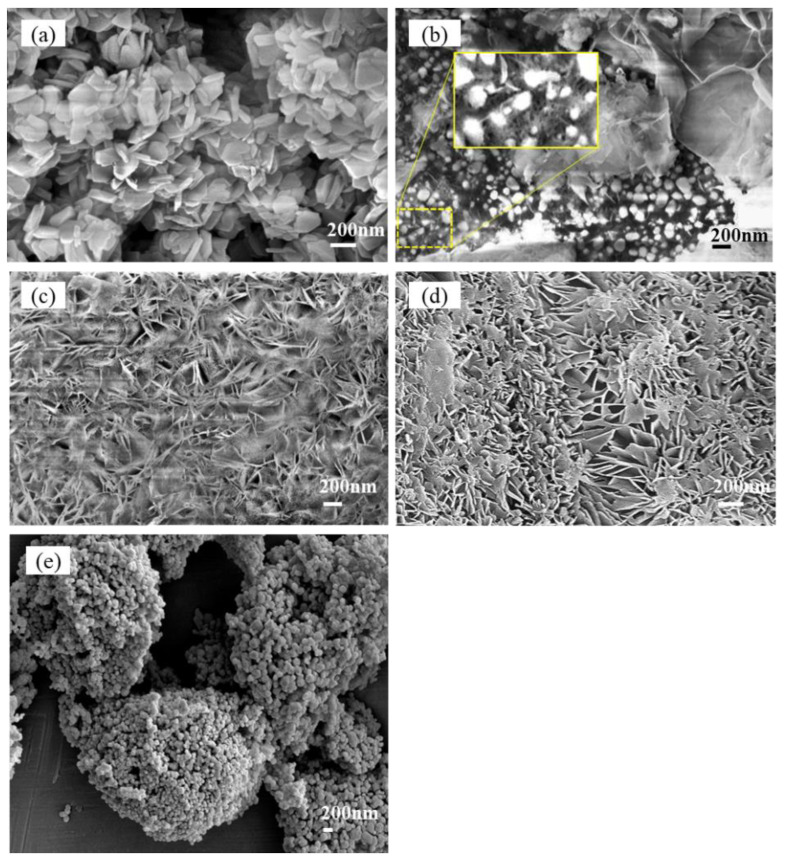
FESEM image of the hardened paste fracture. (**a**) #1-28d (×5000), (**b**) #3-3d (×40,000), (**c**) #3-28d (×40,000), (**d**) #4-28d (×50,000), and (**e**) MgO (×10,000).

Figure 5a shows the microstructural characteristics of the fractured samples with hydration products consisting of pure MgO under a FESEM after 28 days of maintenance in water. The fine plate-like hydration products of different particle sizes were stacked together, where most of the particles were less than 500 nm in size, and the pores between the particles were irregular in shape. Figure 5b shows the microstructural characteristics of the #3-3d sample with the 7.87% Nes addition, and three structures were observed; namely, (1) the interlaced growth of thin sheet-like hydration products with a large number of about 0.5–1 μm pores inside; (2) irregular round-shaped particles of unhydrated MgO with a particle size smaller than 200 nm; (3) cross-shaped lamellar aggregates hidden below the MgO particles, with a large number of nanoscale pores inside (box in Figure 5b). Based on the analysis of the MIP results, the third structure of the #3-3d sample was its main characteristic structure. With a longer curing time of 28 days (sample #3-28d), the third similar texture became the key feature of sample #3. Figure 5c shows the microscopic features of sample #3-28d, which exhibited a third structure similar to sample #3-3d, with interlaced growth of thin lamellar hydration products and the development of nanoscale pores inside. Figure 5d shows the #4-28d sample with the 14.35% Nes addition, which had similar characteristics as the #3-28d sample, with the difference that its lamellar hydration products were thicker and the pores were not uniformly distributed. Figure 5e shows the microstructure characteristics of the unhydrated MgO raw material, irregular round particles with different particle sizes, which is the same as the second structure in Figure 5b below.

### 3.4. XRD Analysis and Phase Characteristics

**Figure 6:**
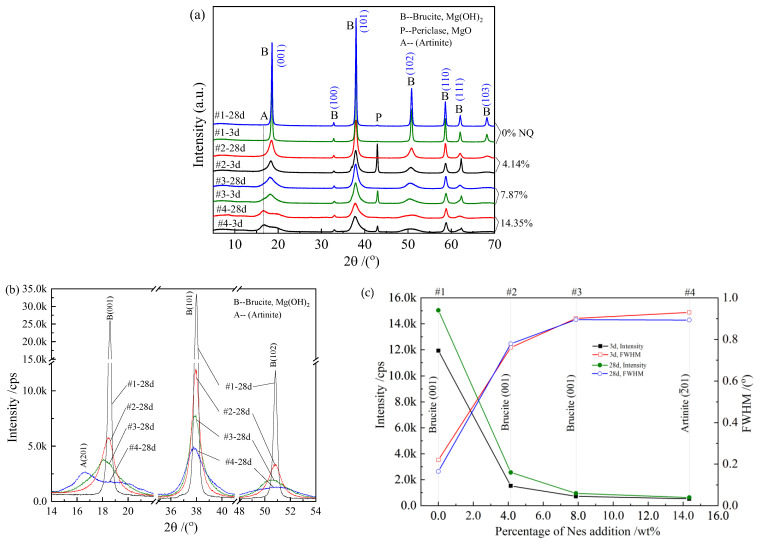
X-ray diffraction pattern of the hardened pastes and diversity of the Mg(OH)_2_ main peaks. (**a**) The XRD patterns of the water-cured samples; (**b**) the comparison of the brucite peaks on the (001), (101), and (102) crystalline planes; (**c**) the intensity of the brucite diffraction peak at (001) and its FWHM (width at half peak) values.

The XRD patterns of the water-cured samples are shown in Figure 6a. For the pure MgO paste (samples #1-3d and #1-28d), the diffraction peaks of brucite Mg(OH)_2_ were narrow and strong, while the diffraction peaks of periclase MgO were very weak, even at a curing time of 3 days. This indicated the high hydration reactivity of MgO as a raw material. As shown in Figure 5a, the hydration product had good crystallinity under the FESEM, which was typical of brucite plate crystals. For the crystals that contained the Nes addition, the brucite peaks became lower and wider, especially in the (001), (101), and (102) lattice planes. In all samples with the Nes addition, the diffraction peaks of the unhydrated MgO were evident in the 3-day-aged samples.

Figure 6b shows the comparison of the brucite peaks on the (001), (101), and (102) crystalline planes of the four samples at a curing time of 28 days. We found that as the percentage of the Nes addition increased, the intensity of the peaks decreased, the peaks became wider, and their corresponding 2θ decreased, or d-spacing increased accordingly. For the #4-28d sample with the 14.35 wt% Nes addition, the peak at (001) was no longer identified, and a diffraction peak close to the artinite ($\overset{—}{2}$01) appeared. The chemical formula of artinite was MgCO_3_·Mg(OH)_2_·3H_2_O, and the ($\overset{—}{2}$01) peak was the second-strongest peak in the JCPDS standard model with 2θ of 16.587°. After subtracting the background diffraction, the intensity of the brucite diffraction peak at (001) and its FWHM (width at half peak) values were plotted, as shown in Figure 6c. As shown in the figure, FWHM increased and gradually stabilized with an increase in Nes addition, and the peak intensity decreased and stabilized with an increase in Nes addition for the 3- and 28-day curing aged samples.

### 3.5. FT-IR Analysis and Group Properties

**Figure 7:**
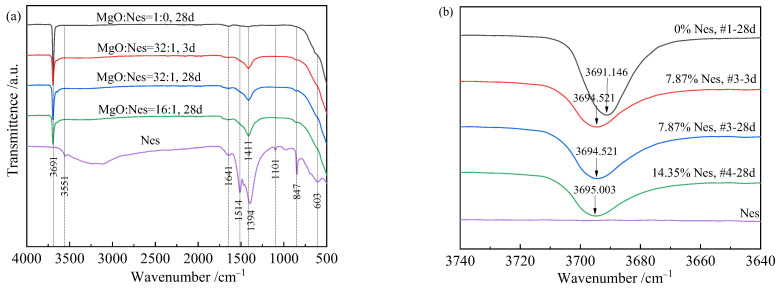
FTIR spectra of the hardened pastes and Nes raw material. (**a**) The four samples and the synthesized Nes; (**b**) local graph at 3691 cm^−1^.

The FTIR spectra of the four samples and the synthesized Nes are shown in Figure 7. The 3691 cm^−1^ band in Figure 7a referred to the antisymmetric O–H stretching vibration of the oxide lattice in Mg(OH)_2_ [33]. This band also appeared in the samples containing Nes, but its peak shifted toward a larger wave number as the Nes addition increased, as shown in Figure 7b. At the same curing time of 28 days, the peak wave number was 3691 cm^−1^ for sample #1 with pure MgO and increased to 3694 and 3695 cm^−1^ for samples #3 and #4 with Nes additions, respectively. For sample #3 with the same Nes content, the corresponding peaks remained constant at 3694 cm^−1^ for curing times of 3 and 28 days. The band at 1411 cm^−1^ was only present in samples #3 and #4 with Nes (Figure 7a). This band was located in the 1400–1420 cm^−1^ region and represented a strong absorption band of the HCO_3_^–^ unit [34]. This was slightly different from the absorption band of the HCO_3_^–^ unit in the Nes raw material at 1394 cm^−1^ [35,36]. The two bands at 847 and 1101 cm^−1^ in the Nes spectrum, representing the absorption band of the CO_3_^2–^ unit, did not appear in samples #3 and #4, as they may have been too weak to detect. Sample #4 shows a weak and small peak at 1092 cm^−1^, which may be Artinite [28]. The information for each absorption peak corresponding to Figure 7 is presented in Table 3.

### 3.6. TG/DSC Analysis and Thermal Effects

**Figure 8:**
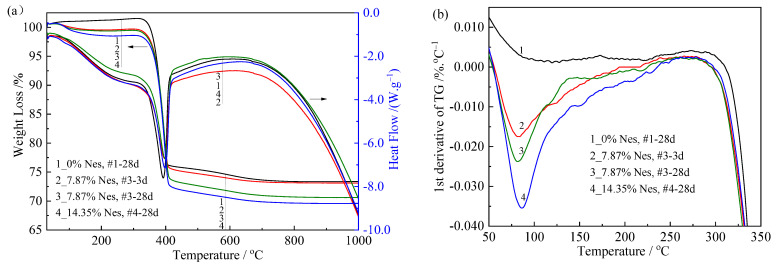
TG/DSC and partial DTG curves for the hardened pastes. (**a**) The TG/DSC curves of the four water-cured samples (The left arrow is the percentage weight loss and the right arrow is Heat Flow); (**b**) the local DTG curves.

The TG/DSC curves of the four water-cured samples are shown in Figure 8a. All samples showed significant weight loss due to Mg(OH)_2_ dehydration. The TG and DSC curves of all samples showed similar morphology, and the decreasing rate of the TG curve indicated a slow-fast-slow trend with increasing temperature. At the same time, the TG curves showed a single-step decreasing trend with a step temperature range of 357–406 °C, and all DSC curves showed a single heat absorption peak at 384–390 °C. Table 4 shows the dehydration temperature range, mass loss, inflection point of the TG curve T_ip_, and a peak temperature of the DSC curve T_p_DSC_ for Mg(OH)_2_. These two temperature values were 390 °C and 384 °C for the Mg(OH)_2_ dehydration process of the #1-28d sample with pure MgO, and the values were 395 °C and 390 °C for the #3-28d sample with the 7.87% Nes addition and 396 °C and 389 °C for the #4-28d sample with the 14.35% Nes addition. This implied that the samples with Nes required higher temperatures than the pure MgO paste to reach a certain dehydration state.

Notably, the TG curves of all samples containing Nes showed slight weight loss in the temperature range of 50–200 °C. Among these, a maximum weight loss of 2.1% was observed in sample #4-28d, while for the pure MgO paste (sample #1), almost no weight change was found in this range. Figure 8b shows the local DTG curves obtained after the differentiation of the TG curves with respect to temperature. We found that the weight loss (DTG peak area) at a temperature of 50–200 °C increased with the amount of Nes addition and the curing time of the samples with a corresponding increase in the peak temperature. The weight loss at this temperature range was due to the effect of dehydration, which meant that the samples containing the Nes additive contained a small amount of water with molecular crystallization.

## 4. Discussion

When the chemically pure, lightly burned MgO with fine particles was mixed with water, the surfaces of the MgO particles started to dissolve. When the concentration of Mg^2+^ and OH^–^ ions in the solution reached supersaturation, the Mg(OH)_2_ crystals precipitated out. With the progression of the hydration process, the mixture coagulated, hardened, and eventually formed a block with a certain strength. Under the wet-air condition, the compressive strength increased with age. However, when the demolded samples were immersed in deionized water, the original dissolution–crystallization process was changed, and Mg(OH)_2_ developed into fine plate-like crystals, which were stacked together (Figure 5a). This structure was detrimental to the strength development of the samples.

The presence of HCO_3_^–^ ions in the mixed solution after the addition of Nes changed the hydration process of MgO and affected the precipitation of the hydration products. This made the total pore volume of the samples larger, and the pore size smaller, with most of the pores consisting of nanopores, and the total pore surface area increased significantly (Table 2). The hydration products showed lamellar–lamellar interlacing growth (Figure 5b–d). The mechanism associated with this phenomenon remains to be explored. The paste still had good strength and high porosity due to the refinement of the pore size and the interlaced growth of the hydration products. XRD analysis showed that the crystal structure of the hydration products in the samples containing Nes was similar to that of brucite Mg(OH)_2_. With the 14.35 wt% Nes addition, the diffraction peak of brucite in the (001) plane was too weak and could not be observed, and a peak similar to that of the artinite ($\overset{—}{2}$01) plane appeared. However, in the FTIR spectrum of sample #4-28d, no symmetric stretching peak at 1092 cm^−1^ [28,38] for the CO_3_^2–^ unit of this indeterminate phase was observed, and no other strong diffraction peaks of the indeterminate were found in the XRD spectrum.

The FTIR spectra of samples #3 and #4 with Nes additions showed a clear absorption peak at the 1411 cm^−1^ position, while sample #1 with pure MgO had no peak. The 1411 cm^−1^ peak corresponded to a strong absorption of HCO_3_^–^, indicating that the hydration product contains HCO_3_^–^ structural units. From the TG and DTG curves, the sample with the Nes addition contained crystalline water that could be removed at low temperatures. The study of the MgO hydration product after Hmgs addition by Kuenzel et al. [28] found that although the XRD spectrum showed low crystallinity Mg(OH)_2_, the combined morphology, FTIR, and Raman spectroscopy results suggested that the hydration product composition should be between Mg(OH)_2_, Hmgs, and H_2_O in one of the phases. Based on the chemical expression of HMC, the hydration product of the Nes addition was written as xMgCO_3_·Mg(OH)_2_·yH_2_O, or against the structural formula of Nes crystals Mg(HCO_3_)(OH)·2H_2_O [37,38], this hydration product could be expressed as x[Mg(HCO_3_)(OH)]·Mg(OH)_2_·zH_2_O. Therefore, the hydration process of MgO with the Nes addition, as well as the total reaction, could be expressed using Equation (1) or Equation (2):

MgO + x[MgCO_3_·3H_2_O] → xMgCO_3_·Mg(OH)_2_·yH_2_O + (3x-y-1) H_2_O (1)

MgO + x[Mg(HCO_3_)(OH)·2H_2_O] → xMg(HCO_3_)(OH)·Mg(OH)_2_·zH_2_O + (2x-z-1) H_2_O (2)

Neglecting the dissolution loss or formation of other phases during hydration, the x-values of the final hydration products of samples #3 (7.87% Nes) and #4 (14.35% Nes) were calculated as 0.025 and 0.048, respectively. Due to the very small x-values, the hydration products showed structures and properties similar to Mg(OH)_2_. With an increase in Nes addition, the peaks of Mg(OH)_2_ in the XRD spectra of the cured paste hydration products showed a tendency to become lower and wider, indicating that the crystals were small and may have been new phases. In this case, the poor X-ray crystallinity was due to either being very thin in the direction perpendicular to the basal plane, or possibly due to some other kind of internal disorder. In the FTIR spectrum, the band at 3691 cm^−1^ changed regularly in the direction of the larger wavenumber.

## 5. Conclusions

(1) The addition of a small amount of Nes also helped to improve the strength of reactive MgO paste under water-curing conditions. The hydration product changed from small plate-like Mg(OH)_2_ crystals to interlaced sheet-like crystals in samples containing Nes.
(2) The porosity increased from 36.25% to 64.55%, and the total pore surface area increased from 4.6 m^2^/g to 118.5 m^2^/g. The total pore volume increased, the pore diameter decreased, and most of the pores were nanopores in the samples with a small amount of Nes addition.
(3) Compared with the pure MgO sample, the hydration product diffraction peak of the sample with the Nes addition became lower and wider. The crystal structure of the hydration products was similar to that of Mg(OH)_2_, with a larger d-spacing at the (001), (101), and (102) planes.
(4) FTIR and TG results showed that the hydration products contained HCO_3_^–^ structural units and a small amount of crystallized water, which could be expressed as xMgCO_3_·Mg(OH)_2_·yH_2_O or x[Mg(HCO_3_)(OH)]·Mg(OH)_2_·zH_2_O. The samples with 7.87% and 14.35% Nes additions had x-values in the final hydration products of 0.025 and 0.048, respectively.

## Figures and Tables

**Table 1:** Correspondence of MgO–Nes molar ratios and their mass fractions.

Sample No.	Designed MgO:Nes Molar Ratio		Water-to-Solid Ratio	Percentage of Nes Addition (wt%)
	MgO	Nes		
#1	1	0	1.1	0
#2	64	1	1.1	4.14
#3	32	1	1.1	7.87
#4	16	1	1.1	14.35

**Table 2:** Pore structure parameter of the hardened pastes.

Sample No.	#1-28d	#2-28d	#3-3d	#3-28d	#4-28d
MgO:Nes molar ratio	1:0	64:1	32:1	32:1	16:1
Nes addition (wt%)	0	4.14	7.87	7.87	14.35
Total pore volume (mm^3^/g)	351.3	473.0	506.1	428.5	1059.6
Total pore area (m^2^/g)	4.6	121.3	111.1	112.7	118.5
Average pore diameter (nm)	303.5	15.6	18.2	15.2	35.8
Dry bulk density (kg/m^3^)	1032	1054	1070	893	609
Porosity (%)	36.25	49.83	54.12	38.27	64.55

**Table 3:** Summary of the bands detected and their origins for the FTIR spectra in Figure 7.

Band Position (cm^−1^)	Origin	Movement	Reference
603	Mg-O	Mg-O bending vibration	[36,37]
847	CO_3_^2−^ from Nes	ν_2_ symmetric bending vibration	[35,36]
1101	CO_3_^2−^ from Nes	ν_1_ symmetric stretching vibration	[35,36]
1394, 1514	CO_3_^2−^/HCO_3_^−^ from Nes	ν_3_ antisymmetric stretching vibration	[35,36]
1411	HCO_3_^−^ from unknown phase		[34]
1641	H_2_O	H-O-H bending vibration	[28]
3551	Nes water of crystallization		[36]
3691~3695	Mg(OH)_2_	Anti-symmetrical O-H stretching vibration of lattice hydroxide	[28,33]

**Table 4:** Thermal dehydration data for the hardened pastes from TG/DSC analyses.

Sample No.	#1-28d	#3-3d	#3-28d	#4-28d
Temperature range of dehydration (°C)	310–602	296–613	293–604	298–631
Inflection point of dehydration by TG (T_ip_) (°C)	390	394	395	396
Peak temperature of dehydration by DSC (T_p_DSC_) (°C)	384	388	390	389
Mass loss of dehydration (%)	27.1	25.9	27.8	28.3
Calculated Mg(OH)_2_ content in hydrated product (wt%)	87.6	83.9	90.1	91.7
Heat absorption of dehydration (J/g)	1074	1116	975	905

## Data Availability

Not applicable.

## References

[1] Rotana H., Kemal C. (2020). Accelerated carbonation of reactive magnesium oxide cement (RMC)-based composite with supercritical carbon dioxide (scCO_2_). J. Clean. Prod..

[2] Oh D.Y., Noguchi T., Kitagaki R., Park W.J. (2014). CO_2_ emission reduction by reuse of building material waste in the Japanese cement industry. Renew. Sustain. Energy Rev..

[3] Almeida M., Ferreira M. (2018). Ten questions concerning cost-effective energy and carbon emission optimization in building renovation. Build. Environ..

[4] Emad B., Ezzatollah S., Muhammad I.R. (2021). Challenges against CO_2_ abatement strategies in cement industry: A review. J. Environ. Sci..

[5] Zhang R.X., Bassim N., Panesar D.K. (2018). Characterization of Mg components in reactive MgO—Portland cement blends during hydration and carbonation. J. CO2 Util..

[6] Walling S.A., Provis J.L. (2016). Magnesia-based cements: A journey of 150 years and cements for the future. Chem. Rev..

[7] Al-tabbaa A. (2013). Reactive Magnesia Cement, Eco-Efficient Concrete Part IV: Future Alternative Binders and Use of Nano and Biotech.

[8] Dung N.T., Hay R., Lesimple A., Celik K., Unluer C. (2021). Influence of CO_2_ concentration on the performance of MgO cement mixes. Cem. Concr. Compos..

[9] Zhang R.X., Arrigoni A., Panesar D.K. (2021). Could reactive MgO cement be a green solution? The effect of CO_2_ mineralization and manufacturing route on the potential global warming impact. Cem. Concr. Compos..

[10] José N., Hawreen A., Miguel B., Luís E., Jorge D.B. (2020). Magnesia (MgO) Production and characterization, and its influence on the performance of cementitious materials: A Review. Materials.

[11] Shand M.A. (2006). The Chemistry Technology of Magnesia.

[12] Unluer C., Al-tabbaa A. (2013). Impact of hydrated magnesium carbonate additives on the carbonation of reactive MgO cements. Cem. Concr. Res..

[13] Unluer C., Al-tabbaa A. (2015). The role of brucite, ground granulated blastfurnace slag, and magnesium silicates in the carbonation and performance of MgO cements. Constr. Build. Mater..

[14] Mo L.W., Deng M., Tang M.S. (2010). Effects of calcination condition on expansion property of MgO-type expansive agent used in cement-based materials. Cem. Concr. Res..

[15] Sonat C., Lim C.H., Liska M., Unluer C. (2017). Recycling and reuse of reactive MgO cements—A feasibility study. Constr. Build. Mater..

[16] Li X., Qiu R., Xue F.B., Fang L., Cheng F.Q. (2020). Effects of unreactive MgO and impurities in light burned MgO on the hydration process and performance of base magnesium sulfate cement. Constr. Build. Mater..

[17] Eubank W.R. (1951). Calcination studies of magnesium oxides. J. Am. Ceram. Soc..

[18] Pachta V., Tsardaka E.C., Stefanidou M. (2021). The role of flame retardants in cement mortars exposed at elevated temperatures. Constr. Build. Mater..

[19] Chang C.G., An L.Y., Lin R., Wen J., Dong J.M. (2022). Effect of Calcination Temperature on Mechanical Properties of Magnesium Oxychloride Cement. Materials.

[20] Bettelini M. (2020). Systems approach to underground safety. Undergr. Space.

[21] Hua N., Tessari A., Khorasani N.E. (2021). Characterizing damage to a concrete liner during a tunnel fire. Tunn. Undergr. Space Technol..

[22] Dung N.T., Unluer C. (2016). Improving the performance of reactive MgO cement-based concrete mixes. Constr. Build. Mater..

[23] Al-tabbaa A., Vandeperre L.J. (2007). Accelerated carbonation of reactive MgO cements. Adv. Cem. Res..

[24] Vlasopoulos N., Cheeseman C.R. (2011). Binder Composition. U.S. Patent.

[25] Vlasopoulos N. (2013). Process of Producing Cement Binder Compositions Containing Magnesium. U.S. Patent.

[26] Dung N.T., Unluer C. (2017). Sequestration of CO_2_ in reactive MgO cement-based mixes with enhanced hydration mechanisms. Constr. Build. Mater..

[27] Dung N.T., Unluer C. (2018). Development of MgO concrete with enhanced hydration and carbonation mechanisms. Cem. Concr. Res..

[28] Kuenzel C., Zhang F., Ferrándiz-Mas V., Cheeseman C.R., Gartner E.M. (2018). The mechanism of hydration of MgO-hydromagnesite blends. Cem. Concr. Res..

[29] Sanna A., Uibu M., Caramanna G., Kuusik R., Maroto-Valer M.M. (2014). A review of mineral carbonation technologies to sequester CO_2_. Chem. Soc. Rev..

[30] Ferrini V., Vito C.D., Mignardi S. (2009). Synthesis of nesquehonite by reaction of gaseous CO_2_, with Mg chloride solution: Its potential role in the sequestration of carbon dioxide. J. Hazard. Mater..

[31] Kloprogge J.T., Martens W.N., Nothdurft L., Duong L.V., Webb G.E. (2003). Low temperature synthesis and characterization of nesquehonite. J. Mater. Sci. Lett..

[32] Ren H.R., Chen Z., Wu Y.L., Yang M.D., Chen J., Hu H.S., Liu J. (2014). Thermal characterization and kinetic analysis of nesquehonite, hydromagnesite, and brucite, using TG-DTG and DSC techniques. J. Therm. Anal. Calorim..

[33] Frost R.L., Kloprogge J.T. (1999). Infrared emission spectroscopic study of brucite. Spectrochim. Acta Part A Mol. Biomol. Spectrosc..

[34] George S. (2004). Infrared and Raman Characteristic Group Frequencies: Tables and Charts.

[35] Frost R.L., Palmer S.J. (2011). Infrared and infrared emission spectroscopy of nesquehonite Mg(OH)(HCO_3_)·2H_2_O—Implications for the formula of nesquehonite. Spectrochim. Acta Part A Mol. Biomol. Spectrosc..

[36] Skliros V., Tsakiridis P., Perraki M. (2020). A combined Raman, Fourier transform infrared, and X-ray diffraction study of thermally treated nesquehonite. J. Raman Spectrosc..

[37] Kandiban M., Vigneshwaran P., Potheher I.V. (2015). Synthesis and characterization of MgO nanoparticles for photocatalytic applications. Natl. Conf. Adv. Cryst. Growth Nanotechnol..

[38] Frost R.L., Bahfenne S., Graham J. (2010). Raman spectroscopic study of the magnesium carbonate minerals—Artinite and dypingite. J. Raman Spectrosc..

